# An enhanced chemiluminescence bioplatform by confining glucose oxidase in hollow calcium carbonate particles

**DOI:** 10.1038/srep24490

**Published:** 2016-04-15

**Authors:** Congmin Wang, Cuisong Zhou, Yuyin Long, Honglian Cai, Cuiyun Yin, Qiufang Yang, Dan Xiao

**Affiliations:** 1 College of Chemistry, Sichuan University, 29 Wangjiang Road, Chengdu 610064, People’s Republic of China; 2 College of Chemical Engineering, Sichuan University, 29 Wangjiang Road, Chengdu 610065, People’s Republic of China

## Abstract

A chemiluminescence (CL) amplification platform based on HCC/Lucigenin&GOx (HLG) film was developed. Hollow structural calcium carbonate (HCC) particles were used as alternative materials for carrying both enzyme and CL reagent. The model enzyme (GOx), immobilized in confined space of HCC particles, exhibited an improved biocatalysis. The Michaelis constant (K_m_) and the enzymatic rate constant (k_cat_) were determined to be 0.209 μM and 2.21 s^−1^, respectively, which are much better than those of either free GOx in aqueous solution or the GOx immobilized on common nanomaterials. Based on the HLG platform, CL signal was effectively amplified and visualized after adding trace glucose, which could be attributed to the HCC particles’ high biocompatibility, large specific surface area, attractive interfacial properties and efficient interaction with analyses. The visual CL bioplatform showed an excellent performance with high selectivity, wide linear range and low detection limit for sensing trace glucose. Because it eliminates the need of complicated assembly procedure and enables visualization by the naked eye, the sensitive and selective CL bioplatform would provide wide potential applications in disease diagnosis and food safety.

Enzyme-relative enhanced chemiluminescence (CL) has been receiving increased interest in various scientific fields, such as environmental monitoring, food analysis and clinical diagnosis^1^. CL, without the need of any excitation light source and any spectral resolving system, has many attractive advantages in low background signals, high sensitivity, wide linear range, simplicity of operation and cost-effectiveness^2^. Nevertheless, CL analysis has to suffer from shortcomings, such as poor selectivity because of co-existing species and relatively low emission intensity resulting from some CL reactions possessing very low efficiency in transforming chemical energy into light^3^. This issue can be resolved by introduction of enzyme, especially immobilized enzyme on some matrixes, such as grapheme oxide, CNT, TiO_2_ nanotubes and PANI microtubes^4^,^5^,^6^,^7^. Due to confine effect provided by these materials, immobilized enzyme can not only guarantee the selectivity of CL reaction but also cause the enhancement of enzymatic reactivity.

Calcium carbonate (CaCO_3_) is not only widely recognized bone filling materials in medicine but also the most popular filler in industrial application^8^. CaCO_3_ particles have some polymorphs mainly including calcite, vaterite and aragonite. Among them, hollow structural calcium carbonate (HCC) particles exhibited amazing encapsulation capability of biomolecules and drugs because of their many beneficial properties such as high porosity, high surface area to volume ratio and biocompatibility towards biomolecules^9^,^10^,^11^. More importantly, their hollow structure has great potential for providing confined space to improve enzyme biocatalysis. So, understanding the enzymatic reaction kinetics occurring within the confine space of HCC particles is of interest and would provide a novel strategy to effectively and specifically amplify CL signal. Nevertheless, to our knowledge, the HCC particles have never been used for any enzyme-enhanced CL amplification.

Herein, we present a facile strategy to develop a novel CL platform based on HCC/Lucigenin&GOx (HLG) film for sensitive detection of glucose in human serum samples. The CL platform was facilely prepared by adding a mixture solution of GOx and lucigenin on HCC film to get a HLG film. When glucose was incubated with the HLG film to generate H_2_O_2_, enzyme reaction kinetic parameters such as enzymatic rate constant kcat and Michaelis-Menten constant (Km) on the CL platform were studied, better than those of free GOx and the immobilized GOx on the reported grapheme oxide CL system. A high sensitivity with a LOD of 9.0 pM and good selectivity were shown. All advantages would be ascribed to the HCC platform that not only provides high ratio of surface-to-volume for better mass and energy transfer, but also gives rise of confine effect for facilitating the maintenance of bioactivity and the high affinity toward substrate of immobilized enzyme. No need of complicated assembly procedure and pretreatment of sample, the sensitive and selective CL platform would provide wide potential applications in disease diagnosis and food safety.

## Results and Discussion

### Design strategy

**Figure 1:**
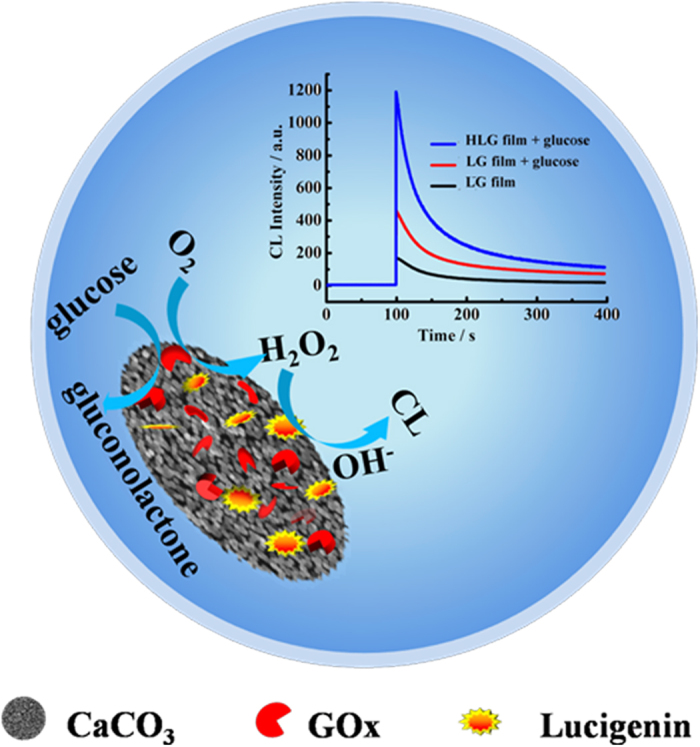
Schematic illustration of the HLG CL platform.

Figure 1 represents the schematic procedure for fabricating the HLG film and the CL amplification strategy for sensing glucose. As a proof-of-concept, HCC particles with unique hierarchical hollow structure are prepared using a reported starch template^12^. Due to porosity structure and high interfacial area of HCC particles, both GOx and lucigenin can be efficiently entrapped via physical adsorption. It was supposed that confine effect of the porous structural HCC particles might maintain even and improve biocatalysis of the immobilized enzyme. Furthermore, the porous structural HLG platform has a large geometric surface area, which might be in favor of efficient interaction between the immobilized molecules (like GOx or CL regent) and analytes. In the present of glucose, H_2_O_2_ can be generated because of the GOx biocatalyst reaction happening on the HLG film. After the injection of NaOH, CL signal can be detected due to CL reaction of lucigenin with H_2_O_2_. Owing to the enhanced enzyme bioactivity caused by confine effect of HCC, CL signal can be amplified effectively, which would result in a sensitive and specific detection of glucose. Consequently, glucose concentration can be sensitively quantified owing to amplified CL response. The facile and novel CL biosensing platform based on the HLG film would have potential applications including disease diagnosis and food safety.

### Characterization

**Figure 2:**
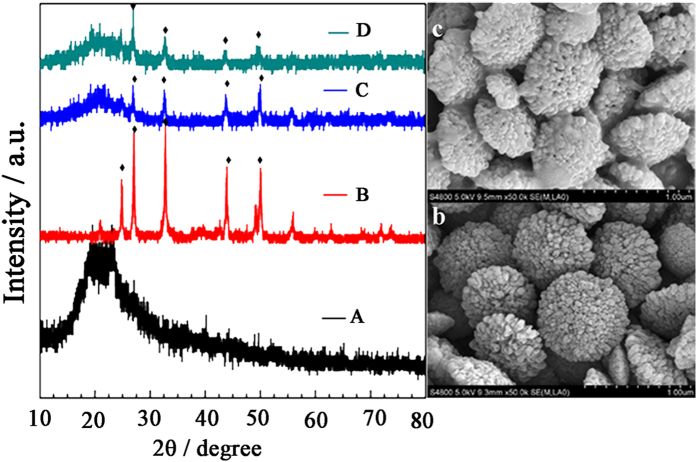
SEM images of HCC film (b) and HLG film (c). X-ray diffraction pattern of GOx (**A**), HCC particles (**B**), HLG film before (**C**) and after (**D**) CL reaction.

SEM image shows that the HCC particles have an average diameter of about 550 ± 50 nm and exhibit a porous spherical morphology (Fig. 2b). It is ascribed to soluble starch, used as a template molecular, can couple the crystallization of CaCO_3_ nanoparticles and *in situ* symmetry-breaking assembly of these crystallites into hollow spherical shells^12^. Consequently, plenty of defects and pores are observed on the HCC surface. The HCC particles maintained their porous structure well even after a coating process using GOx and lucigenin solution (Fig. 2c), which was consistent with XRD results.

Characteristic diffraction peaks of vaterite polymorph, such as (1 1 0), (1 1 2), (1 1 4), (3 0 0) and (1 1 8) were observed for both HCC film and HLG film (Fig. 2B,C). It is worth noting that the HLG film maintained vaterite polymorph and GOx well (Fig. 2D). This indicates that the HCC particles of the HLG film not only keep a stable hollow structure but also provide good vessels to hold GOx. N_2_ adsorption-desorption isotherms were further used to measure specific surface area of the HCC particles. After the loading of GOx and lucigenin, their specific surface area effectively decreased from 64.64 to 42.23 (m^2^/g) (Fig. S1). A conclusion can be made that the hollow structural HCC can not only be a potential holder for carrying GOx and lucigenin but also act as nanoscaled-enzymatic reactors.

### Enhanced and visualized CL signal of HLG film

**Figure 3:**
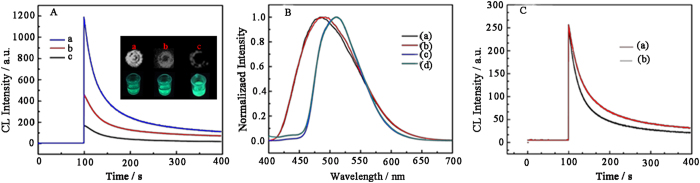
(**A**) CL kinetic curves of HLG incubated with 100 nM glucose (a) and LG incubated with (b) or without (c) 100 nM glucose. The inset shows the corresponding photographs of the CL signal (up) and FL signal (bottom). (**B**) CL spectra of lucigenin-H_2_O_2_ (a) and HLG (b); FL spectra of lucigenin (c) and HCC/lucigenin (d). (**C**) CL kinetic curves of lucigenin-H_2_O_2_ (a) and HCC/lucigenin-H_2_O_2_ (b). Concentration of NaOH is 0.12 M.

To verify the improved CL response of the HLG film for sensing glucose, the CL behavior of lucigenin&GOx (LG) film with or without HCC particles were compared. As shown in Fig. 3A, when the LG film was incubated with 100 nM glucose for 40 min, strong CL signal was observed after the addition of 0.12 M NaOH (Fig. 3A,B). This is because the immobilized GOx catalyze the oxidation of glucose to generate H_2_O_2_ that further reacts with lucigenin at in alkaline condition to produce CL emission^13^. Interestingly, for the HLG platform, its CL signal was increased 3 times higher than that of the LG film on the same experimental condition (Fig. 3A–C). It is worth noting that either these CL response or FL response for sensing low concentration glucose (100 nM) can be visualized using a simple FluorChem M system (inset of Fig. 3A). Obviously, the CL response for sensing glucose was apparently improved by the introduction of the HCC particles. There might be two reasons to explain the CL-enhancement phenomena. One is that the lucigenin-H_2_O_2_ CL reaction has enhanced emission intensity because the HCC matrix might facilitate the transformation of chemical energy into light^3^. The other one might be that the HCC matrix can provide a nanoscale reactor to get an enhanced emzyme biocatalysis due to the confine effect. Therefore, the FL behavior of lucigenin-H_2_O_2_ system and HLG film were investigated under the same experimental conditions. As shown in Fig. 3B, both this maximum emission wavelengths are at 480 nm, whatever HCC particles present (d) or not (c). It indicates the CL emitter of the HLG film is still excited state N-methylacridone (NMA)^13^. Meanwhile, the CL emission efficiencies of the lucigenin-H_2_O_2_ system with (a) or without HCC particles (b) were the same (Fig. 3C). Obviously, the HCC particles are not capable of improving emission efficiency of the lucigenin CL system, but enhance the biocatalysis of GOx, which is also supported by previous reports^14^,^15^.

### The enzymatic reaction kinetics of immobilized GOx in the HLG reactor

**Figure 4:**
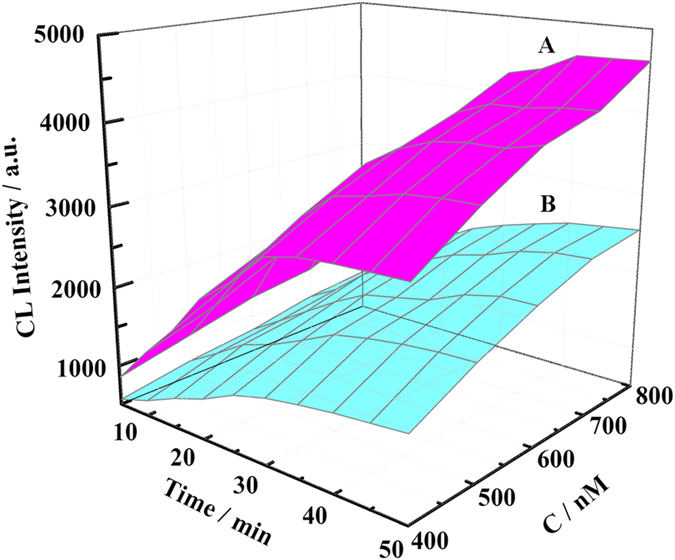
The CL spectra of the HLG (**A**) and LG (**B**) film after incubation with different concentrations of glucose at different reaction time. The concentration of glucose is 400 nM, 500 nM, 600 nM, 700 nM and 800 nM, respectively.

The kinetics of the enzyme reaction in the HLG reactor were studied in details, by monitoring the CL response triggered by immobilized enzyme. Figure 4 shows that in the present of glucose, the CL intensity increases with the increasing reaction time up to 40 min, and then reaches a maximum value. The Km, determining the affinity of the enzyme for the substrate, was calculated using Lineweaver-Burke plot^16^,^17^. The Km of the GOx immobilized on HLG film was calculated to be 0.209 μM, much lower than that of immobilized GOx without HCC particles (0.759 μM). The kcat of HLG was calculated to be 2.21 s^−1^, much better than those of LG (1.06 s^−1^). This is supported that the excellent biocompatibility and confine space of HCC particles can provide a favorable microenvironment for GOx to retain its bioactivity and keep high binding affinity to the substrate glucose^18^.

### Analytical performance for detecting glucose

**Figure 5:**
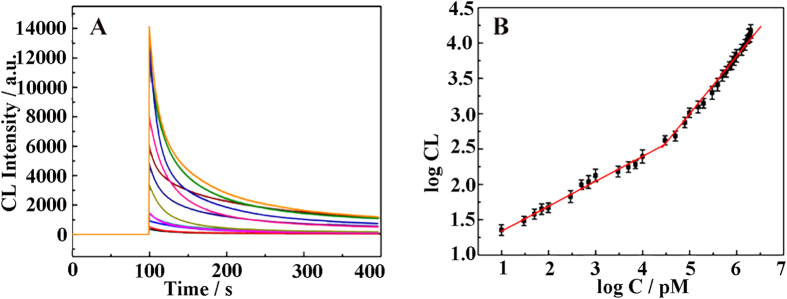
CL kinetic curves for HLG film to various glucose solutions in 0.12 M NaOH aqueous. (**A**) The linear relationship between the relative CL response and the concentration of glucose shows two wide linear ranges from 0.01 nM to 50.0 nM and 50.0 nM to 2.0 μM, respectively (**B**).

To fabricate a biosensor having an excellent analytical performance, some key parameters were optimized including reaction time, NaOH concentration, lucigenin concentration and the amount of HCC particles in details, which were 40 min, 0.12 M, 200 μM and 0.50 mg mL^−1^, respectively (Fig. S2). Especially, CL intensity dramatically increases as increasing of HCC particles from 0.10 to 0.50 mg mL^−1^, and gets the highest signal at 0.50 mg mL^−1^. This would be attributed to the good enzyme carrier that the HCC particles act where more HCC particles can carry more enzyme molecule. However, when the concentration is so higher than 0.50 mg mL^−1^, the HCC film would be too thick to have porous structure, which inhibits the interaction with analyses. Under the optimized condition, the analytical performance of the biosensor was assessed using various concentrations of glucose. As Fig. 5 shown, the CL response increased as the concentration of glucose increased. The good linear relationship between the logarithm of CL intensity and the logarithm of glucose concentration was observed. There are two wide linear ranges from 0.01 nM to 50.0 nM and 50.0 nM to 2.0 μM. Their linear equations log I = 1.153 + 0.895 log C (r = 0.9887) and log I = 2.032 + 0.351 log C (r = 0. 0.9849). I is CL intensity and C is the concentration of glucose (pM). The limit of detection (LOD) was calculated as 9.0 pM according to LOD = 3σ/S, where σ is the standard deviation and S is the slope of the calibration curve equation. It is better than that of previous reports^19^,^20^,^21^.

### Selectivity against interferences and long-term stability of the biosensor

**Figure 6:**
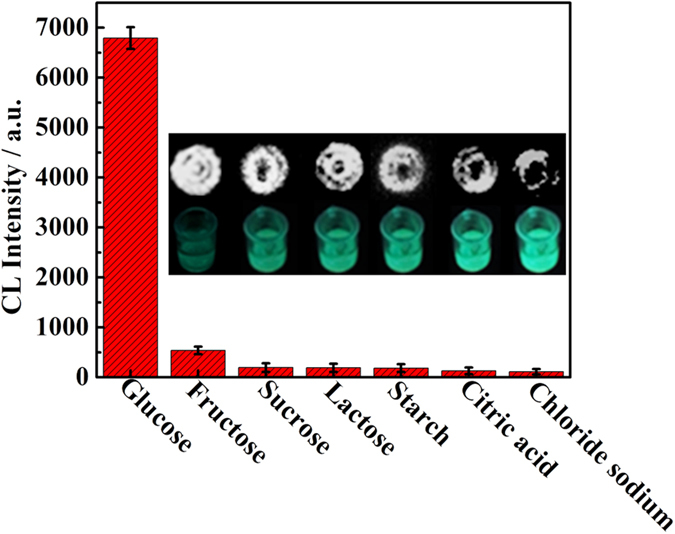
Interference test of the CL biosensor based on HLG film. The concentration of glucose is 1.0 μM. Concentration of each interference is 10.0 μM. The inset shows the corresponding photographs of the CL signal (up) and FL signal (bottom).

The practical utility of a sensor often depends upon its selectivity. Therefore, some potential substances were tested one by one to evaluate the selectivity of the glucose biosensor. Their concentrations were 10.0 μM. As shown in Fig. 6, when 1.0 μM glucose solution was added, a significant enhancement of the CL intensity was observed. However, when high concentration interferences (10.0 μM) were tested, any notable response from the biosensor was not observed. An excellent selectivity the CL biosensor based on HLG film for glucose was demonstrated. Such good selectivity was also visualized depending on the CL imaging (inset of Fig. 6).

With the aim of investigating its long-term stability, the HLG film was stored at 4 °C in a refrigerator, and its CL response was tested at intervals. Initially, no obvious decrease of the CL intensity was observed. After storage for 20 days and even one month, the absorbance retained 96.4% and 90.6% of its original value, respectively (Fig. S3). The bioactivity of immobilized GOx and lucigenin were well maintained, which indicated that the HCC film provided a biocompatible microenvironment for biomolecules.

### Determination of glucose in human serum sample

To illustrate the feasibility of the HLG film in biologically relevant matrix, it was employed to detect glucose in human blood serum. In this work, human blood serum was simply diluted with an appropriate dilution ratio to yield testing sample solutions. The recovery test was conducted. All the data are summarized in Table 1. These satisfied recovery results demonstrated that the HLG film offer an excellent, accurate, and precise method for the determination of glucose in a biologically relevant matrix. Importantly, these two different concentrations of glucose can be visualized in human blood serum (Fig. S4).

### Application of visualization detection based on both CL and FL imaging

**Figure 7:**
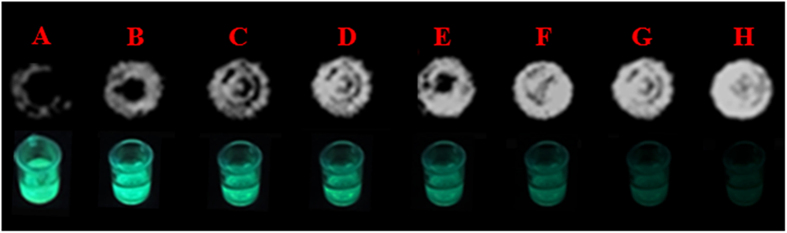
Photograph of CL signal (up) and FL signal (bottom) of the HLG film-based CL platform for sensing glucose. The concentration of glucose is 0 nM (**A**), 0.1 nM (**B**), 1.0 nM (**C**), 10.0 nM (**D**), 100.0 nM (**E**), 500.0 nM (**F**), 1.0 μM (**G**) and 2.0 μM (**H**). Concentration of NaOH is 0.12 M.

Visual glucose detection has become increasingly important for the laboratory and point-of-care diagnostic applications because it eliminates the need for sophisticated instrumentation and enables visualization by the naked eye. Such detection methods are economical to implement and feature simplicity and portability. In this work, dual-signals including CL and FL imaging were used to visualize glucose at a low concentration. As shown in Fig. 7, when the HLG film was incubated with different concentrations of glucose, CL imaging was captured using a FluorChem M technique after the addition of 0.12 M NaOH. The corresponding FL imaging of the HLG film after the CL reaction was recorded using a digital camera under a UV irradiation. As the concentration of glucose increased from 0.1 nM to 2.0 μM, a visualized CL response increased while luminescence signal gradually fade (Fig. 7). To amplify the visualization detection, “ΔI_CL_ + |ΔI_FL_|” is used as the response signal to quantitatively determine the concentration of glucose. It can be observed that the “ΔI_CL_ + |ΔI_FL_|” also increases linearly with the increase of the glucose corresponding range from 0.1 to 1.0 μM and the corresponding linear function is (ΔI_CL_ + |ΔI_FL_|) = 21.99 log C (pM) – 10.36 (R^2^ = 0.9743). The LOD is 1.0 nM (S/N = 3) (Fig. S5), comparable to or even better than most colorimetric glucose sensors^22^,^23^.

## Conclusion

In this paper, we present a facile strategy to develop a novel CL platform based on HLG film for visualizing low concentration of glucose. CL data demonstrated that the entrapped enzyme exhibited its improved biocatalysis that resulted in enhanced CL signal, which might be ascribed to the confine effect of hollow structural HCC particles. Two wide detection ranges of 0.01–50 nM and 50 nM–2.0 μM were obtained. The CL platform based on HLG film exhibited a good sensitively with LOD of 9.0 pM (S/N = 3). It is worth noting that a dual-signaling amplification strategy based on “ΔI_CL_ + |ΔI_FL_|” can be developed to visualize low concentration of glucose at nanomolar level. No need of complicated assembly procedure and pretreatment of sample, the high sensitive and high selective CL platform would provide wide potential applications in disease diagnosis and food safety.

## Experimental

### Materials

Lucigenin was purchased from Tokyo Chemical Industry Co. Ltd. (Tokyo, Japan). Glucose oxidase (GOx) and Nafion were obtained from Sigma-Aldrich (St. Louis, USA). Sodium carbonate, calcium chloride, soluble starch, glucose, lactose, sucrose, fructose, citric acid, hydrogen peroxide (H_2_O_2_) and sodium hydroxide were purchased from Chengdu Chemicals (Sichuan, China). All reagents were of analytical grade and used as received without further purification. All aqueous solutions were prepared using deionized distilled water.

### Measurements and apparatus

CL signal was recorded using a model IFFS-A multifunctional chemiluminescence system (Ruimai Electronic Science Co, China). CL spectra and fluorescence (FL) spectra were measured by a model F-7000 Fluorescence Spectrophotometer (Hitachi, Japan). The morphology of HCC particles were charactered by SEM (Hitachi S-4800, Japan). The crystal structure of HCC particles were analyzed by a Tongda TD-3500 XRD with Cu-Ka radiation (λ = 0.15 nm) operating at 30.0 kV and 20.0 mA (Liaoning, China). The Brunauer-Emmett-Teller (BET) specific surface area was obtained from the nitrogen (N_2_) adsorption method using a Quadrasorb SI Automated Surface Area (Quantachrome, USA). The CL images were charactered by a FluorChem M (ProteinSimple, USA). The FL images were recorded using a digital camera under irradiation a model ZF-20 D UV spectrophotometer (Henan, China).

### Preparation of HCC particles

The HCC particles were synthesized according to a reported method^12^. In brief, two very dilute solutions were vigorously mixed at 30 °C for ten minutes. One is Na_2_CO_3_ solution (2 mM, 110 mL). The other one is 120 mL of 1.67 mM CaCl_2_ solution containing soluble starch (0.042 wt%) used as an additive.

### Fabrication of HCC/Lucigenin&GOx film

HLG film was prepared as follows: The HCC colloidal suspension (0.5 mg mL^−1^) was prepared by dispersing HCC particles in deionized water and keeping ultrasonically treatment for 10 min. GOx was also dissolved in deionized water with a concentration of 10 mg mL^−1^. 100 μL of the HCC colloidal suspension (0.5 mg mL^−1^) was added to a well of a polystyrene 96-well plate. Dried for 30 min at 50 °C, a porous HCC film was formed. 40 μL lucigenin/GOx mixture solution with a defined concentration was added on the HCC film, and then further dried for 30 min at 30 °C. Finally, 10 μL Nafion (0.5 wt%) was coated on the film and dried for 20 min at 30 °C to obtain the HLG film.

## Additional Information

**How to cite this article**: Wang, C. *et al*. An enhanced chemiluminescence bioplatform by confining glucose oxidase in hollow calcium carbonate particles. *Sci. Rep*. **6**, 24490; doi: 10.1038/srep24490 (2016).

## Supplementary Material

Supplementary Information

## Figures and Tables

**Table 1:** Determination and recovery of glucose in real samples.

Sample	Glucose added (μM)	Glucose found (μM)	Recovery %	RSD (n = 3) %
Blood serum	0.0100	0.0103	103	3.20
Blood serum	1.00	0.989	98.9	2.10
