# Supplementary material for: An enhanced chemiluminescence bioplatform by confining glucose oxidase in hollow calcium carbonate particles
Source: Sci Rep. 2016 Apr 15;6:24490. doi: 10.1038/srep24490 (PMC4832249; doi:10.1038/srep24490)
Supplement: Supplementary Information [file srep24490-s1.pdf]

Supporting Information

An enhanced chemiluminescence bioplatform by confining glucose oxidase in hollow calcium carbonate particles

Congmin Wang[1], *Cuisong Zhou*[1,*], *Yuyin Long*[1], *Honglian Cai*[1], *Cuiyun Yin*[1], *Qiufang Yang*[1], *Dan Xiao* [1,2,*]

[1]College of Chemistry, Sichuan University, 29 Wangjiang Road, Chengdu 610064, People's Republic of China
[2]College of Chemical Engineering, Sichuan University, 29 Wangjiang Road, Chengdu 610065, People's Republic of China

***** To whom correspondence should be addressed

E-mail: zcs@scu.edu.cn (C. S. Zhou)

E-mail: xiaodan@scu.edu.cn

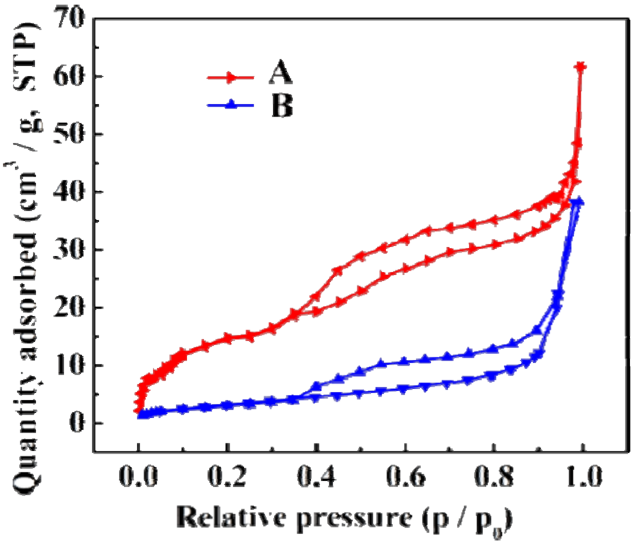

Supplementary Figure S1. Nitrogen adsorption-desorption isotherm of HCC particles before **(A)** and after loading lucigenin and GOx **(B)**.

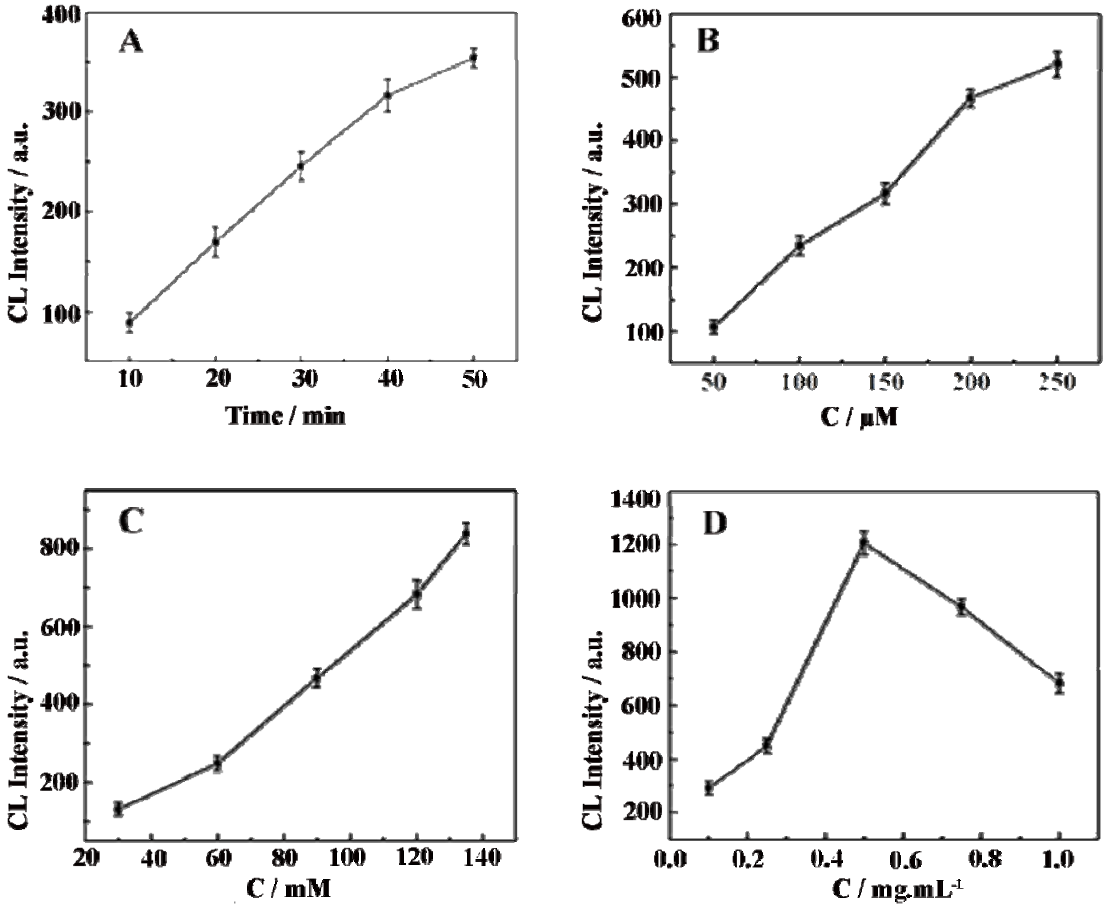

Supplementary Figure S2. The optimization of the reaction time **(A)**, the concentration of lucigenin **(B)**, the concentration of NaOH **(C)** and the concentration of HCC **(D)**.

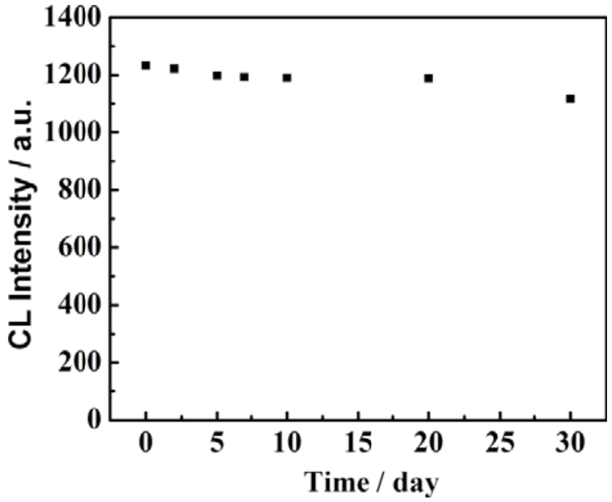

Supplementary Figure S3. The stability of the HLG film in 0.12 M NaOH for detecting 100 nM glucose.

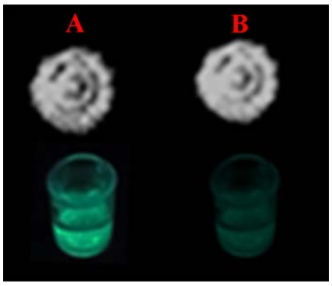

Supplementary Figure S4. Photograph of CL signal (up) and FL signal (bottom) of the HLG film-based CL platform for sensing glucose in human blood serum. The concentration of glucose is 10.0 nM (A) and 1.0 μM (B), respectively.

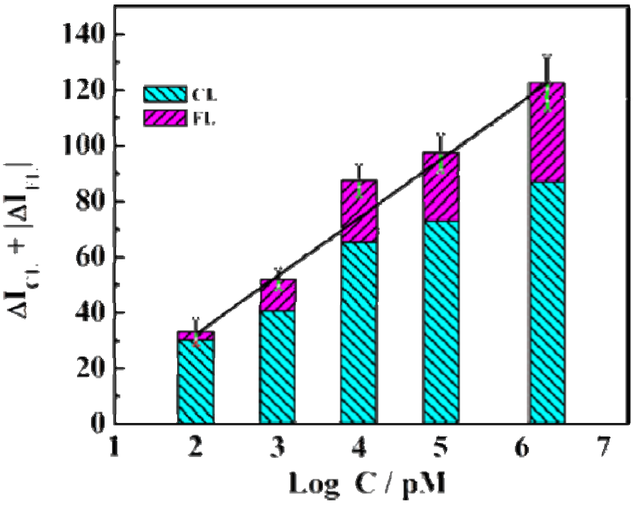

Supplementary Figure S5. The linear working curve for visualizing glucose based on "$\Delta I_{CL} + |\Delta I_{FL}|$" as the response signal. The linear function is ($\Delta I_{CL} + |\Delta I_{FL}|$) = 21.99 Log C (pM) – 10.36 (R^2 = 0.9743) with a linear range from 0.1 nM to 1.0 μM. The LOD is 1.0 nM (S/N = 3)
